# Primarily Gasless Robot-Assisted Cholecystectomy in Dogs: A Cadaveric Feasibility Study

**DOI:** 10.3390/vetsci13030292

**Published:** 2026-03-20

**Authors:** Francisco M. Sánchez-Margallo, Mauricio Veloso Brun, Juan A. Sánchez-Margallo

**Affiliations:** 1 Scientific Direction, Jesús Usón Minimally Invasive Surgery Center, 10171 Cáceres, Spain; 2 Department of Small Animal Clinics (DCPA), Universidade Federal de Santa Maria (UFSM), Santa Maria 97105-900, Brazil; mauriciovelosobrun@hotmail.com; 3 Bioengineering and Health Technologies Unit, Jesús Usón Minimally Invasive Surgery Center, 10171 Cáceres, Spain; jasanchez@ccmijesususon.com

**Keywords:** robot-assisted surgery, Primarily gasless laparoscopy, cholecystectomy, Versius surgical system, minimally invasive surgery, canine cadaver model

## Abstract

Gallbladder disease is a common cause of morbidity in dogs, and cholecystectomy remains the definitive treatment in many cases. Although minimally invasive approaches offer recognized advantages, their adoption is limited by technical complexity and by the physiological effects of carbon dioxide (CO₂) pneumoperitoneum. This cadaveric study evaluated the feasibility of performing primarily gasless robot-assisted cholecystectomy under abdominal wall suspension in five canine cadavers. A normobaric operative field was established using a suspension device combined with the VersiusTM robotic platform. “Primarily gasless” was operationally defined as abdominal wall suspension without continuous pneumoperitoneum, allowing short-duration low-pressure CO₂ insufflation (≤8 mmHg) exclusively as a rescue maneuver when exposure was insufficient. Surgical feasibility was assessed through structured case-level reporting, including docking time, operative time, exposure quality of the hepatocystic triangle (predefined ordinal scale), clipping feasibility, intraoperative events, instrument exchanges, and need for rescue CO₂ insufflation. All procedures were completed in the cadaveric specimens. In three cadavers, the procedure was performed entirely without CO₂. In two cadavers, a single short-duration low-pressure CO₂ insufflation (6–8 mmHg for 3–5 minutes) was applied as a rescue maneuver. Adequate or optimal visualization of the hepatocystic triangle was achieved in all cases, and no intraoperative injuries occurred. These findings support the technical feasibility of a primarily gasless robotic approach in a canine cadaveric model. Controlled in vivo studies are required to evaluate the physiological impact, perioperative outcomes, and translational applicability before clinical implementation.

## Simple Summary

Gallbladder disorders are increasingly diagnosed in dogs, and many affected patients require surgery to prevent severe complications such as bile leakage or sepsis. Traditionally, gallbladder removal has been performed through an open approach, but this technique is associated with considerable risks and a difficult recovery. In recent years, less invasive procedures, including laparoscopy and robot-assisted surgery, have been explored to improve patient outcomes. In this study, we evaluated a new combination of techniques: performing cholecystectomy with a robotic platform under gasless abdominal suspension. This method avoids the use of carbon dioxide to create working space, which may reduce stress on fragile patients, while robotics appeared to facilitate precise dissection and favorable working conditions in this experimental setting. The procedure was tested successfully in canine cadavers, with consistent access to key anatomical structures, and no technical complications were observed during the procedures in this cadaveric model. These findings support further investigation of this hybrid approach in canine biliary surgery.

## 1. Introduction

Gallbladder disease has become an increasingly recognized cause of morbidity in dogs. Although biliary sludge is more frequently identified on ultrasonography, gallbladder mucocele (GBM) represents the most clinically significant and surgically relevant biliary disorder [1,2]. While some affected dogs remain subclinical until advanced stages, others present with nonspecific signs that may rapidly progress to severe complications such as rupture, bile peritonitis, or septic shock [3,4,5]. In such situations, cholecystectomy is considered the treatment of choice and remains the definitive therapy [6,7].

Open cholecystectomy has been the standard approach in veterinary practice. However, perioperative mortality rates in dogs are consistently higher than those reported in human medicine, particularly in the presence of rupture or systemic compromise [2,8]. Complications such as bile leakage, sepsis, pancreatitis, and coagulopathies remain major contributors to poor outcomes, particularly in older patients or those with concurrent endocrine and systemic disease [9,10,11].

In canine surgery, minimally invasive approaches have been progressively developed to reduce morbidity associated with hepatobiliary procedures (Table 1). Laparoscopic cholecystectomy is feasible in both experimental and clinical canine studies, with reported benefits including reduced pain, faster recovery, and superior visualization of biliary anatomy [6,12]. Nevertheless, the laparoscopic technique presents several challenges, including a steep learning curve, conversion rates of up to 30% in some reports, and a risk of bile duct injury [13,14,15].

Reliance on carbon dioxide (CO_2_) pneumoperitoneum to create the working space represents an additional concern. Insufflation can impair venous return, reduce pulmonary compliance, induce hypercapnia, and alter splanchnic circulation, effects that may be poorly tolerated in fragile patients [16,17]. Moreover, prolonged exposure to pneumoperitoneum has been associated with postoperative adhesions and altered inflammatory responses in experimental models [18].

To overcome these physiological drawbacks, gasless laparoscopy may represent a potential alternative approach that warrants further evaluation in clinical studies. Lifting devices that elevate the abdominal wall allow safe exposure without insufflation and have been successfully tested in veterinary applications, such as diaphragmatic hernia repair and renal biopsy [19,20]. Although this approach offers clear advantages in high-risk patients, it may reduce cranial working space and complicate smoke evacuation [16].

Robot-assisted surgery has recently expanded into veterinary medicine, providing high-definition three-dimensional (3D) imaging, enhanced precision, and improved ergonomics through articulated instruments [21,22,23]. The integration of advanced imaging modalities, such as near-infrared fluorescence cholangiography with indocyanine green (ICG), further enhances intraoperative visualization of biliary anatomy, potentially increasing safety in complex cases [24,25].

**Table 1:** Minimally invasive and adjunct intraoperative techniques relevant to canine cholecystectomy.

Author (Year)	Approach	Study Type	Number of Cases	Main Findings
Mayhew et al. (2008) [6]	Standard multiport laparoscopy	Clinical	6 dogs	Feasible laparoscopic cholecystectomy in dogs with gallbladder mucocele.
Scott et al. (2016) [13]	Standard multiport laparoscopy	Clinical	20 dogs	Laparoscopic cholecystectomy in 20 dogs with a 30% conversion rate to open surgery.
Simon & Monnet (2020) [12]	Single-port laparoscopy (SILS)	Clinical	15 dogs	Single-port laparoscopic cholecystectomy using a single-port access system (SPAS).
Kondo et al. (2023) [7]	Subserosal dissection laparoscopic cholecystectomy	Clinical	34 dogs	Subserosal layer dissection as a laparoscopic cholecystectomy technique in dogs.
Kim & Lee (2024) [25]	Near-infrared fluorescence cholangiography (ICG-guided intraoperative imaging)	Clinical	27 dogs	Near-infrared ICG cholangiography for biliary anatomy and CBD patency assessment.

The present study describes, for the first time, the performance of gasless robot-assisted cholecystectomy in canine cadavers using the Versius^TM^ surgical robot (CMR Surgical, Cambridge, UK). By combining the physiological advantages of gasless laparoscopy with the precision of robotic systems, this work aims to establish a foundation for further clinical research and eventual application in veterinary patients.

## 2. Materials and Methods

### 2.1. Study Design

This cadaveric experimental study was designed to evaluate the technical feasibility of performing primarily gasless robot-assisted cholecystectomy in dogs. All procedures were carried out at the Jesús Usón Minimally Invasive Surgery Center (CCMIJU; Cáceres, Spain), following internal ethical and biosafety standards for the use of animal cadavers in surgical research.

### 2.2. Specimens

Five adult Beagle cadavers with no history or gross evidence of hepatobiliary disease were obtained through a teaching and research donation program. Cadavers included three males and two females, with body weights ranging from 11.8 to 13.4 kg. Animals originated from routine donations to the anatomy program and had not undergone previous abdominal surgery. Cadavers were refrigerated and used within 24 h of euthanasia to minimize tissue degradation. Before each procedure, abdominal integrity and biliary anatomy were confirmed by exploratory inspection.

### 2.3. Robotic Platform and Instrumentation

All procedures were performed using the Versius^TM^ robotic surgical system (CMR SurgicalCambridge, UK). The system consisted of a surgeon console with high-definition 3D vision and three robotic arms (one for the camera and two for surgical instruments). Articulated wristed instruments (Maryland bipolar forceps, monopolar hook, monopolar scissors, and needle drivers) were employed. When required, standard 5- and 10-mm laparoscopic instruments were employed (grasper, suction-irrigation device, clip appliers).

For this study, the term “primarily gasless” was operationally defined as completion of the procedure under abdominal wall suspension without continuous pneumoperitoneum, permitting short-duration low-pressure CO_2_ insufflation (≤8 mmHg) exclusively as a rescue maneuver when mechanical elevation alone did not provide sufficient exposure. Continuous pneumoperitoneum was not maintained at any stage of the procedure.

Abdominal access without CO_2_ insufflation was achieved by means of a gasless suspension system (VET 9000 Platform; BhioSupplyEsteio, RS, Brazil). This device, originally described by Brun et al. in 2022 [19], was subsequently refined and further developed through collaboration between the CCMIJU and associated engineering teams [19]. The system consists of a subxiphoid retractor connected to an external lifting frame, enabling elevation of the anterior abdominal wall to establish a stable operative field under ambient pressure. This approach provided adequate exposure for hepatobiliary dissection while avoiding the physiological alterations associated with pneumoperitoneum.

The Versius^TM^ system was configured with a three-arm setup: one 10-mm 3D camera arm positioned centrally, and two robotic arms equipped with 6.8-mm wristed instruments in a triangulated configuration (Supplementary Materials, Figure S1). Arm spacing was optimized to minimize external collision while maintaining internal instrument articulation within the right cranial abdominal quadrant.

### 2.4. Surgical Procedure

Cadavers were maintained in a neutral horizontal position throughout the procedure, without Trendelenburg or reverse-Trendelenburg tilt. This configuration allowed evaluation of surgical exposure and instrument maneuverability under the robotic suspension system without additional positional assistance. A 10-mm camera port was introduced through a transumbilical midline incision. Two 10-mm working ports were placed under direct vision in a triangulated configuration in the right cranial abdomen: one approximately 3–4 cm caudal to the right costal arch along the paramedian line, and the second positioned to achieve ergonomic triangulation toward the hepatocystic triangle while minimizing external arm collision. A separate 5-mm assistant port was positioned laterally in the right cranial quadrant to permit suction-irrigation, clip application, and supplementary retraction when required. Robotic arms were docked individually, and instrument positioning was adapted to allow ergonomic access to the hepatobiliary region. In cases of suboptimal exposure under suspension alone, a standardized stepwise troubleshooting sequence was followed before considering rescue CO_2_ insufflation. This included: (1) optimization of gallbladder fundus traction; (2) adjustment of liver retraction and robotic arm angulation; (3) verification of adequate abdominal wall lift and modification of lift vector when necessary; and (4) smoke evacuation using intermittent suction. Short-duration low-pressure CO_2_ insufflation was considered only if these measures failed to provide adequate visualization of the hepatocystic triangle.

The gallbladder was exposed by gentle retraction of the liver lobes. The hepatocystic triangle was dissected using monopolar scissors under 3D visualization. The cystic duct and artery were individually isolated and ligated with polymer clips (Hem-o-lok^®^; Teleflex Medical, Research Triangle Park, NC, USA). The gallbladder was then dissected from its hepatic fossa using electrocautery, with particular care to preserve the continuity of the common bile duct. The specimen was extracted through the umbilical port.

### 2.5. Data Collection

Primary feasibility endpoints were: (1) unambiguous identification of the cystic duct and artery; (2) secure clipping and division of these structures; and (3) completion of cholecystectomy without macroscopic intraoperative adverse events.

Secondary endpoints included the analysis of docking time, total operative time, exposure grade, need for rescue CO_2_ insufflation, number of instrument exchanges, workflow interruptions, and qualitative intraoperative observations regarding instrument handling and surgeon comfort.

For each procedure, the following parameters were recorded: docking time (from robotic positioning to full instrument readiness); total operative time (skin incision to specimen extraction); quality of exposure of the hepatocystic triangle; feasibility of cystic duct and artery clipping; intraoperative events; number of robotic instrument exchanges; and need for rescue measures (including CO_2_ insufflation or additional assistant maneuvers). Exposure of the hepatocystic triangle was graded using a predefined ordinal scale:

- Optimal: Complete visualization of the cystic duct and artery without the need for rescue measures.
- Adequate: Critical structures identified clearly but requiring minor adjustments (e.g., modified traction or brief rescue CO_2_ insufflation).
- Inadequate: Inability to clearly identify critical structures without major deviation from the protocol.

All procedures were video recorded and independently reviewed by two surgeons with experience in minimally invasive and robotic veterinary surgery. Exposure grading and procedural assessment were performed independently using the predefined criteria. In cases of discrepancy, consensus was achieved through joint video review and discussion. Video-scored variables included hepatocystic triangle exposure grade, clarity of cystic duct and artery identification, need for rescue measures (including CO_2_), instrument exchanges, internal or external collisions, workflow interruptions, and any visible adverse events.

At completion of each procedure, the hepatobiliary region was systematically inspected for macroscopic bile duct injury, vascular damage, clip displacement, or gallbladder perforation. In addition, post-procedure exploratory dissection of the hepatoduodenal ligament region was performed to confirm the integrity of the extrahepatic bile duct.

### 2.6. Statistical Analysis

Given the exploratory nature of the study and the limited sample size, results were analyzed descriptively. Continuous variables were expressed as median (range), whereas categorical outcomes were reported as absolute frequency. No formal statistical comparisons were performed.

### 2.7. Ethical Considerations

This study exclusively used cadaveric specimens and therefore did not require formal approval from an animal care and use committee. All procedures adhered to the guidelines of European Directive 2010/63/EU on the protection of animals used for scientific purposes.

## 3. Results

### 3.1. Procedural Feasibility and Exposure

**Figure 1:**
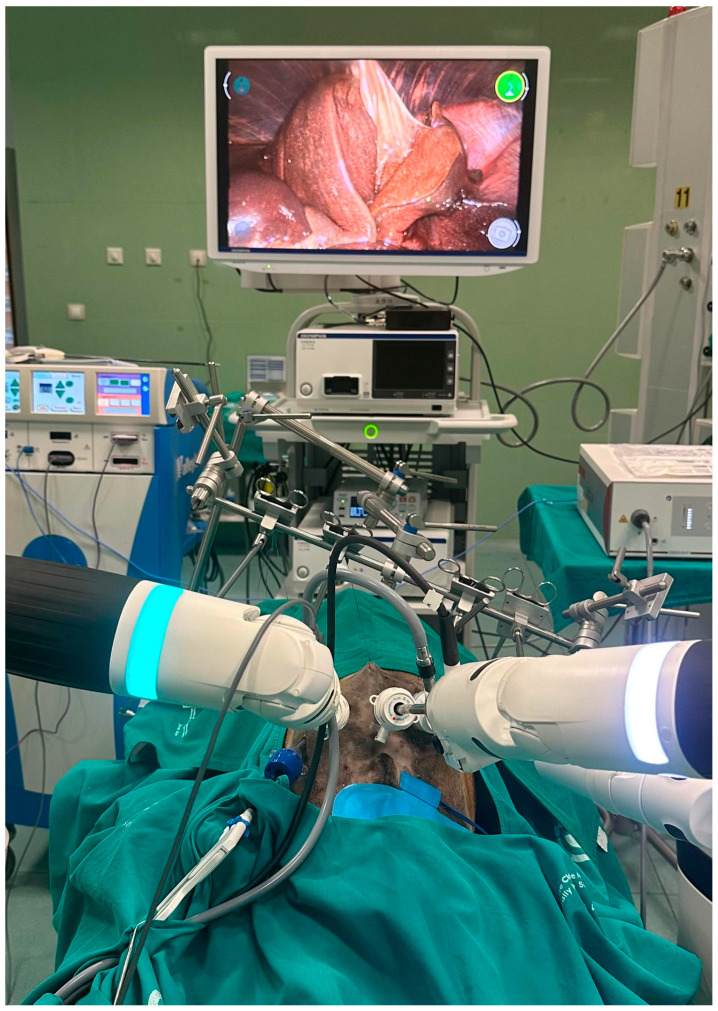

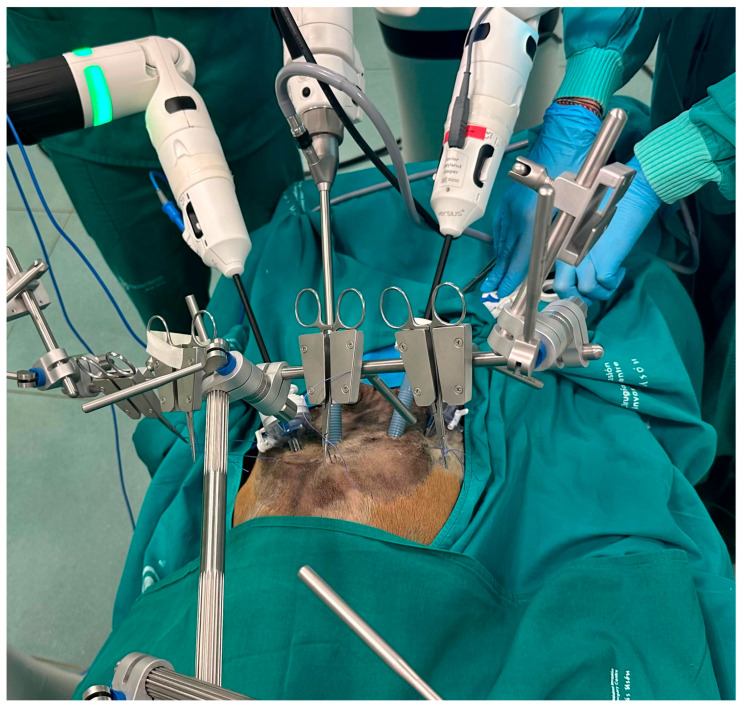
Schematic representation of the abdominal suspension system combined with the Versius^TM^ robotic platform. The anterior abdominal wall is elevated using the suspension device [19]. The working instruments and assistant port were oriented toward the right cranial quadrant, with the camera introduced through a transumbilical midline port.

All five robotic cholecystectomy procedures were completed under primarily gasless conditions using abdominal wall suspension (Figure 1). None of the cases required conversion to an open approach. In three cadavers, the surgical field was maintained entirely under mechanical elevation without CO_2_ insufflation. In two cadavers, a single short-duration low-pressure CO_2_ insufflation (6–8 mmHg for 3–5 min) was applied as a rescue maneuver to enhance cranial exposure and facilitate visualization of the hepatocystic triangle. Traditional laparoscopic cholecystectomy in dogs typically employs pneumoperitoneum pressures ranging from 10 to 12 mmHg [16,17]; thus, the rescue insufflation applied in this study was substantially lower than standard clinical pressures. Continuous pneumoperitoneum was not used in any case. In both instances, rescue insufflation was required during the exposure phase to improve cranial retraction before definitive dissection of the hepatocystic triangle. Suspension lift was first optimized by adjusting the traction vector and abdominal wall elevation; CO_2_ was introduced only after these mechanical adjustments failed to provide adequate visualization.

In all cases, the cystic duct and artery were identified without ambiguity, and complete removal of the gallbladder was achieved. Structured case-level outcomes are summarized in Table 2. No intraoperative complications, such as bile duct injury or gallbladder rupture, were observed.

### 3.2. Operative and Docking Times

Docking of the Versius^TM^ platform was consistently efficient, with a median docking time of 9 min (range 8–10 min). Total operative times, measured from skin incision to gallbladder removal, ranged from 29 to 36 min, with a median duration of 32 min. Operative times in the gasless group tended to be slightly shorter than those in cadavers where low-pressure CO_2_ was briefly applied, although the sample size precluded statistical analysis. Phase-specific analysis suggested that exposure and hepatocystic triangle dissection required approximately 10–15 min, clipping of the cystic duct and artery 3–5 min, and gallbladder bed dissection 12–18 min.

### 3.3. Surgical Exposure and Visualization

**Figure 2:**
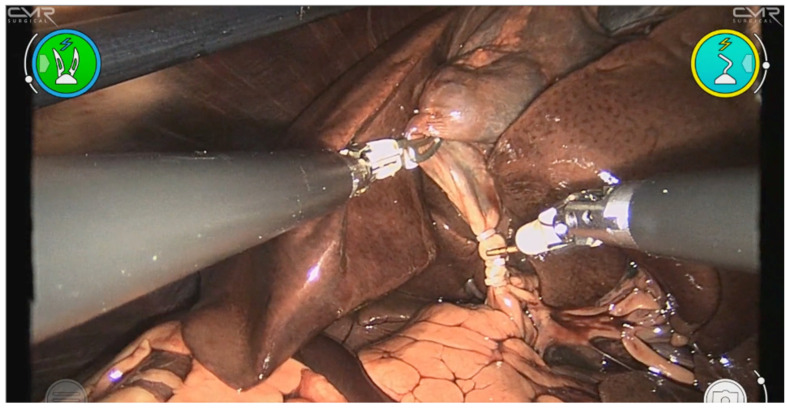
Visualization of the hepatocystic triangle during gasless robot-assisted cholecystectomy. Adequate exposure was consistently obtained despite the absence of pneumoperitoneum.

Adequate exposure of the hepatocystic triangle was achieved in all cadavers (Figure 2). In purely gasless procedures, traction on the gallbladder fundus and mechanical lift of the abdominal wall provided a clear operative field; however, cranial viscera did not fall away to the same extent as under pneumoperitoneum. When smoke accumulation impaired visualization, intermittent suction effectively restored image clarity. The robotic high-definition 3D endoscope provided stable images with enhanced depth perception, facilitating accurate identification of anatomic landmarks and tissue planes, even under the reduced working space inherent to gasless surgery.

Both cases requiring rescue CO_2_ were graded as “Adequate” exposure rather than “Optimal,” suggesting that limited working space under pure suspension was associated with reduced visualization quality. No CO_2_ rescue was required in cases graded as “Optimal.”

### 3.4. Dissection, Clipping, and Instrument Handling

Critical structures were safely clipped using 5-mm polymer Hem-o-lok^®^ clips applied through the assistant port. No clip failures or slippages were observed. Dissection of the gallbladder from the liver bed was achieved without bile leakage or parenchymal tearing. The articulated robotic instruments proved advantageous for maneuvering in the narrow canine subhepatic space, allowing controlled angulation and consistent traction without internal or external instrument collision.

The absence of a robotic clip applier required temporary exchange of one robotic instrument for a laparoscopic clip applier in each case (range: 1–2 exchanges per procedure). Each exchange resulted in an estimated workflow interruption of approximately 2–4 min. These exchanges did not result in technical errors, loss of visualization, clip misplacement, or iatrogenic tissue injury but introduced measurable operative pauses. Future iterations of this approach may benefit from the integration of robot-compatible vessel sealing or energy-based technologies, which could reduce instrument exchanges and further streamline the workflow.

### 3.5. Qualitative Ergonomic Observations

Qualitative intraoperative observations suggested satisfactory instrument maneuverability and working space using the robotic suspension system. The open-console design of the Versius^TM^ system enabled a comfortable seated position with a neutral posture. Articulated instruments appeared to facilitate precise dissection in confined areas. A schematic representation of the surgeon’s seated position at the Versius^TM^ console has been included (Supplementary Materials, Figure S2) to illustrate ergonomic posture and console configuration.

### 3.6. Intraoperative Complications

No intraoperative complications were recorded. Specifically, there were no cases of bile duct injury, gallbladder perforation, or procedure abandonment. The only deviations from the gasless protocol were the two instances of temporary low-pressure CO_2_ use, which were considered technical adjustments rather than complications. All gallbladders were retrieved intact, confirming no macroscopic procedural complications during the procedures.

## 4. Discussion

The present study provides the first description of primarily gasless robot-assisted cholecystectomy in dogs using abdominal suspension in a preclinical cadaveric setting. All procedures were completed successfully, with adequate visualization of the hepatocystic triangle, safe dissection, and effective clipping of the cystic duct and artery. These findings support the technical feasibility of the approach and justify further controlled in vivo investigation.

In canine clinical practice, gallbladder disease is an increasingly important cause of morbidity, with gallbladder mucocele being the most frequently reported biliary disorder [1,2,15]. Although medical therapy may be effective in selected cases, cholecystectomy remains the treatment of choice in patients at risk of rupture or with advanced disease [3,4,5,14]. Traditional open cholecystectomy has been widely practiced, but it is associated with considerable morbidity and mortality, particularly in complicated cases involving bile peritonitis or systemic compromise [8]. Outcomes in these scenarios often reflect the severity of underlying conditions, with perioperative mortality rates ranging from 20% to over 40% [10,26].

The adoption of minimally invasive surgery has progressively transformed hepatobiliary practice. Laparoscopic cholecystectomy, though initially confined to experimental work, is feasible in both clinical and cadaveric studies, with potential advantages such as reduced postoperative discomfort, shorter recovery times, and superior visualization of biliary anatomy [6,7,12]. Clinical data also suggest improved outcomes when performed in stable patients, although bile leakage, hemorrhage, and the need for conversion remain relevant risks [13]. The learning curve and technical difficulty continue to represent major barriers to widespread implementation [27,28].

A major limitation of conventional laparoscopy is the dependence on CO_2_ pneumoperitoneum. While insufflation provides an excellent surgical field, it has been shown to alter venous return, compromise pulmonary compliance, and increase systemic CO_2_ absorption, which can be poorly tolerated in patients with comorbidities or biliary rupture [16,17]. These physiological effects are of particular concern in older animals or those with concurrent endocrinopathies or cardiovascular disease [11]. In addition, prolonged exposure to pneumoperitoneum has been associated with inflammatory changes and the risk of adhesion formation [18].

Primarily gasless laparoscopy has been proposed as an alternative to minimize these drawbacks (Table 3). Abdominal wall lifting systems avoid the hemodynamic consequences of CO_2_ insufflation and have been successfully applied in experimental canine models, including diaphragmatic hernia repair [19] and renal biopsy [20]. While suspension offers a safer physiological profile, limitations include reduced cranial working space and challenges in smoke evacuation [16]. In the present series, these difficulties were mitigated by robotic articulation and controlled suction.

Robot-assisted surgery has recently entered the veterinary field, offering enhanced ergonomics, tremor elimination, 3D visualization, and articulated wristed instruments that replicate the dexterity of the human hand [21,23] (Table 3). These features make robotic platforms particularly suitable for confined anatomical regions such as the hepatocystic triangle. Previous studies have demonstrated the feasibility of robotic procedures in veterinary patients, including experimental intestinal strictureplasty [21], radical prostatectomy [22], and cholecystectomy in canine cadavers with the da Vinci^®^ system (Intuitive Surgical, Inc., Sunnyvale, CA, USA) [23]. It should also be acknowledged that the operating surgeon in the present study had extensive prior experience in advanced laparoscopic hepatobiliary procedures and robotic surgery. This expertise likely contributed to the efficiency observed in docking and operative times and may limit the extrapolation of these results to less-experienced teams. Although favorable ergonomic conditions were observed during the procedures, this study did not include a formal ergonomic assessment using validated scoring systems. Future studies should incorporate objective ergonomic evaluation tools better to characterize surgeon workload and comfort during robotic suspension cholecystectomy.

Recent innovations further expand the possibilities of minimally invasive biliary surgery. Three-dimensional laparoscopy has been shown to improve accuracy and safety in dissection [29]. The integration of near-infrared fluorescence imaging with indocyanine green (ICG) allows real-time mapping of biliary structures, which may reduce the risk of bile duct injury and facilitate intraoperative decision-making [24,25]. These imaging modalities could be particularly beneficial when combined with robotic platforms, where enhanced visualization aligns with precise instrument control. Single-port and subserosal dissection techniques also represent innovative alternatives for canine cholecystectomy [7,12], although their use remains restricted to highly experienced centers due to technical challenges.

**Table 3:** Experimental and innovative minimally invasive techniques relevant to abdominal surgery in dogs.

Author (Year)	Technique	Model	Main Outcomes
Brun et al. (2022) [19]	Gasless laparoscopy for diaphragmatic hernia repair	Canine cadaver	Adequate exposure with suspension device; feasible alternative to pneumoperitoneum
Fransson et al. (2015) [16]	Lift laparoscopy vs. CO_2_ pneumoperitoneum	Live dogs	Gasless approach showed reduced cardiopulmonary stress and lower pain response
Milech et al. (2023) [20]	Gasless laparoscopic renal biopsy	Canine cadaver	Feasible; adequate visualization and specimen collection
Buote et al. (2023) [23]	Robot-assisted cholecystectomy (da Vinci^®^Intuitive Surgical, Inc., Sunnyvale, CA, USA)	Canine cadaver	Feasible; longer docking time; one gallbladder tear reported
Schlake et al. (2020) [22]	Robot-assisted radical prostatectomy	Clinical (dog)	First veterinary application in oncology; safe resection achieved
Sonoda et al. (2007) [21]	Robot-assisted intestinal strictureplasty	Experimental (dog, survival study)	16 procedures; survival confirmed; technically demanding
Azuma & Monnet (2024) [29]	3D vs. 2D laparoscopy for cholecystectomy	Canine cadaver	3D improved precision and safety during dissection

From a clinical perspective, survival outcomes following cholecystectomy have improved in recent years, particularly when surgery is performed electively before rupture [9,14]. Thus, less invasive and more precise surgical strategies are essential to improving safety in high-risk patients. Primarily gasless robot-assisted approaches may represent an interesting technical strategy; however, their potential physiological advantages must be confirmed in controlled in vivo studies.

Despite these encouraging feasibility results, several limitations must be explicitly acknowledged. First, all procedures were performed in healthy cadaveric specimens with normal hepatobiliary anatomy. Clinical cases of gallbladder mucocele, rupture, or inflammatory biliary disease frequently present with tissue friability, adhesions, hemorrhage risk, and distorted anatomy that may significantly increase procedural complexity. Therefore, the present cadaveric model likely underestimates the technical challenges encountered in clinical hepatobiliary pathology.

Second, cadaveric experimentation precludes assessment of intraoperative bleeding control, cardiopulmonary stability, inflammatory response, and postoperative recovery. Consequently, the physiological advantages commonly attributed to gasless surgery cannot be evaluated in this model and require controlled in vivo validation.

Additionally, the extremely small sample size (n = 5) inherently limits statistical interpretation and generalizability. This series should therefore be interpreted strictly as a preliminary technical feasibility study rather than as evidence of comparative efficacy. Further studies using larger sample sizes and clinical cases will be necessary to validate these findings.

In summary, the integration of gasless suspension and robotic assistance represents an experimental minimally invasive strategy in veterinary hepatobiliary surgery. By addressing both the physiological risks of pneumoperitoneum and the ergonomic challenges of laparoscopy, this hybrid approach warrants further investigation as a minimally invasive strategy for canine biliary surgery. Continued research, incorporating clinical validation, adjunct imaging modalities, and development of robot-compatible instruments, will be essential for translating this experimental approach into routine practice.

## 5. Conclusions

Primarily gasless robot-assisted cholecystectomy using the Versius^TM^ platform was technically feasible in a canine cadaveric model. The combination of abdominal wall suspension and robotic articulation allowed consistent identification of critical biliary structures, with only limited short-duration CO_2_ rescue in selected cases. These findings provide a structured foundation for further investigation in controlled in vivo settings. In vivo studies are required to determine the physiological impact, procedural safety in diseased tissue, and comparative performance before any clinical application can be considered in veterinary patients.

## Figures and Tables

**Table 2:** Case-level structured feasibility outcomes for primarily gasless robot-assisted cholecystectomy.

Cadaver	Docking Time (min)	Operative Time (min)	Exposure Grade	Rescue CO_2_ Insufflation (Pressure; Duration)	Instrument Exchanges (*n*)	Protocol Deviations
1	10	29	Optimal	No	1	None
2	9	31	Adequate	6 mmHg; 3 min	2	Brief rescue CO_2_ insufflation
3	9	32	Optimal	No	1	None
4	8	36	Adequate	8 mmHg; 5 min	2	Brief rescue CO_2_ insufflation
5	9	33	Optimal	No	1	None

## Data Availability

The original contributions presented in this study are included in the article/Supplementary Material. Further inquiries can be directed to the corresponding author.

## Supplementary Materials

The following supporting information can be downloaded at https://www.mdpi.com/article/10.3390/vetsci13030292/s1. Supplementary Materials captions: Figure S1: Schematic representation of the experimental setup for robotic abdominal surgery using the Versius™ system. Figure S2: Surgeon operating at the Versius™ open console during a robotic procedure.

## Abbreviations

The following abbreviations are used in this manuscript:

3D	Three-dimensional
GBM	Gallbladder mucocele
CO_2_	Carbon dioxide
CBD	Common bile duct
ICG	Indocyanine green
