# Supplementary material for: Primarily Gasless Robot-Assisted Cholecystectomy in Dogs: A Cadaveric Feasibility Study
Source: Vet Sci. 2026 Mar 20;13(3):292. doi: 10.3390/vetsci13030292 (PMC13029909; doi:10.3390/vetsci13030292)
Supplement: Supplementary file 1 [file vetsci-13-00292-s001.zip › vetsci-4136288-supplementary.pdf]

Supplementary Material

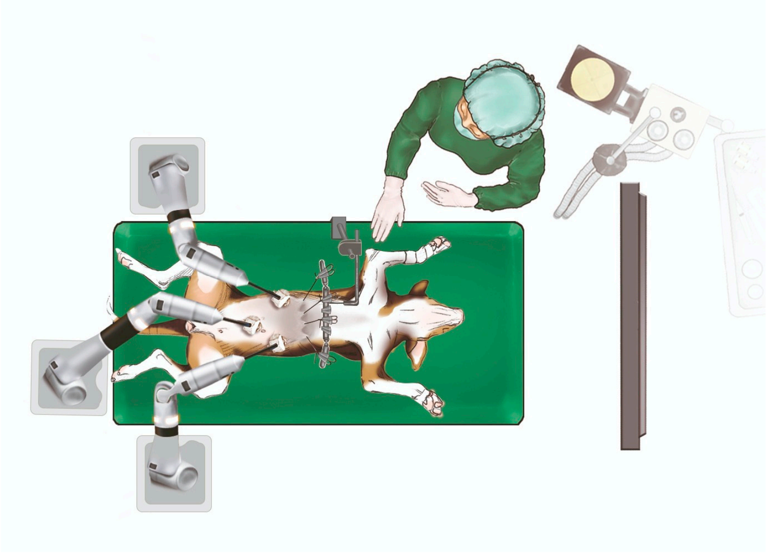

Figure S1. Schematic representation of the experimental setup for robotic abdominal surgery using the Versius™ system. The platform was configured with three robotic arms: a centrally positioned 10-mm 3D camera arm and two 10-mm working arms arranged in a triangulated configuration. The spacing between the arms was optimized to minimize external collisions while preserving adequate internal instrument articulation within the right cranial abdominal quadrant.

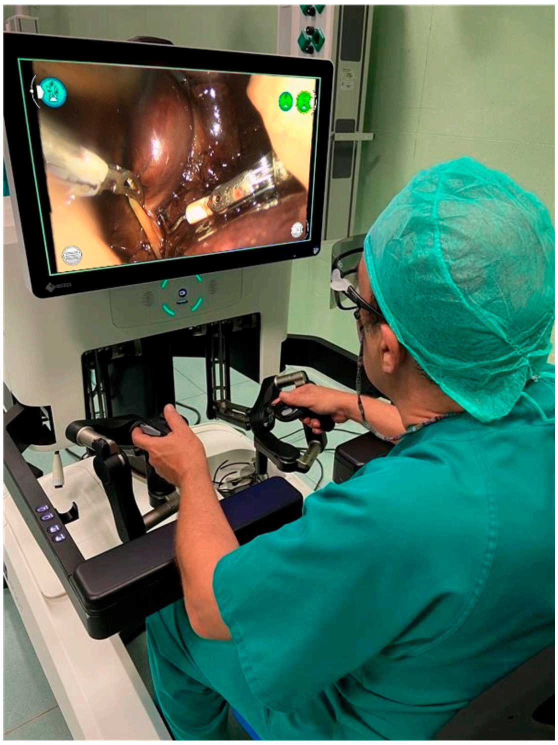

Figure S2. Surgeon operating at the Versius™ open console during a robotic procedure. The system allows a comfortable seated position with a neutral ergonomic posture, reducing physical strain during surgery. The console provides independent control of the robotic camera and articulated instruments, enabling precise manipulation and stable exposure without the need for an assistant to hold the laparoscope. The image illustrates the ergonomic configuration of the surgeon at the console and the real-time operative view displayed on the monitor.
